# Chemical Composition and Acetylcholinesterase Inhibitory Activity of the Volatile Fraction from the Ecuadorian Moss *Sphagnum magellanicum* Brid

**DOI:** 10.3390/molecules31213797

**Published:** 2026-10-22

**Authors:** James Calva, Juan Francisco Dávila, Eduardo Valarezo, Ángel Benítez

**Affiliations:** 1 Departamento de Química, Universidad Técnica Particular de Loja, Loja 110107, Ecuador; bevalarezo@utpl.edu.ec; 2 Carrera de Ingeniería Química, Universidad Técnica Particular de Loja, Loja 110107, Ecuador; jfdavila7@utpl.edu.ec; 3 Biodiversidad de Ecosistemas Tropicales-BIETROP, Herbario HUTPL, Departamento de Ciencias Biológicas y Agropecuarias, Universidad Técnica Particular de Loja (UTPL), Calle Paris s/n y Praga, Loja 110107, Ecuador; arbenitez@utpl.edu.ec

**Keywords:** *Sphagnum magellanicum*, volatile fraction, GC-MS, phytol, acetylcholinesterase, moss

## Abstract

The volatile fraction (VF) of the aerial parts of *Sphagnum magellanicum* Brid. (Sphagnaceae), collected in southern Ecuador (2740 m a.s.l), was obtained for the first time by steam distillation using a Dean–Stark adapted apparatus and characterized by gas chromatography coupled to mass spectrometry (GC-MS) and flame ionization detection (GC-FID). A total of 89 compounds representing 95.28 ± 1.95% of the total VF were identified. The predominant chemical classes were n-alkanes (39.94%), alcohols (17.56%), sesquiterpene hydrocarbons (13.10%), and oxygenated sesquiterpenes (6.48%). Phytol was the major single compound (16.09 ± 0.07%), followed by n-tricosane (9.25 ± 0.82%), n-heneicosane (9.17 ± 0.54%), hexacosane (7.30 ± 0.99%), nonacosane (5.42 ± 0.14%), (Z)-caryophyllene (4.08 ± 0.32%), hexadecanoic acid (3.64 ± 0.25%), and dotriacontane (2.93 ± 0.21%). The VF was evaluated in vitro for acetylcholinesterase (AChE) inhibitory activity using the Ellman colorimetric assay, yielding a moderate inhibitory effect with an IC_50_ value of 77.0 ± 1.03 µg/mL. These results provide the first phytochemical and pharmacological characterization of *S. magellanicum* from Ecuador, establishing it as a potential source of bioactive volatile constituents with potential relevance for further pharmacological investigation.

## 1. Introduction

Mosses belong to the division Bryophyta and comprise over 13,000 described species, making them the second most diverse group of land plants after angiosperms [1,2]. They are widely distributed across all continents, with particular abundance in humid and shaded environments of the Northern Hemisphere [2,3]. The genus *Sphagnum* is recognized for a notable diversity of secondary metabolites, including sterols, triterpenoids (such as ursolic acid), fatty acids, fatty alcohols, and phenolic compounds [4,5]. Chemical characterization studies using gas chromatography coupled to mass spectrometry (GC-MS) have revealed metabolic profiles that vary seasonally, highlighting the presence of carbohydrates, phytosterols, and diterpenes that contribute to the biological properties of these organisms [6].

The volatile fraction of bryophytes has received growing attention as a source of biologically active constituents [7,8]. The identification of volatile compounds in mosses through simultaneous distillation–extraction techniques has revealed the presence of aldehydes and terpenoids, which are key constituents of the volatile fraction of these organisms [9,10]. Studies on *Sphagnum* species specifically have documented the use of in vitro cultures to stabilize and characterize these chemical profiles, confirming the presence of characteristic volatiles in the genus [11]. The phytochemical characterization of the VF of *S. magellanicum* from South American habitats remains largely unexplored, representing a significant knowledge gap in the bryophyte chemistry literature.

The inhibition of acetylcholinesterase (AChE) constitutes a primary therapeutic strategy for the management of Alzheimer’s disease (AD) and related neurodegenerative disorders [12,13]. AChE catalyzes the hydrolysis of the neurotransmitter acetylcholine (ACh) in cholinergic synapses; its inhibition increases ACh availability and improves cholinergic transmission, which is clinically beneficial in early and moderate stages of AD [12]. Current FDA-approved AChEIs, including donepezil, rivastigmine, and galantamine, provide only symptomatic relief and are associated with adverse effects that limit long-term use, thus motivating the search for novel plant-derived inhibitors with improved safety profiles [13,14]. In this context, volatile fractions from aromatic plants and their terpenoid constituents have shown AChE inhibitory capacity, which in some studies has been suggested to involve synergistic interactions among constituents [15,16]. Monoterpenes and sesquiterpenes commonly found in plant volatile fractions, such as α-pinene, limonene, 1,8-cineole, and caryophyllene, have been specifically identified as active agents in AChE inhibition [17,18].

Ecuador possesses one of the highest bryophyte diversities in the world [19], representing a vast reservoir of bioactive compounds with antibacterial, antioxidant, and antiproliferative properties [20]. In the regional context, bryophytes have also attracted attention for their potential cholinesterase-related activity. A survey of 39 bryophyte species representing 16 families detected cholinesterase activity in 30 species from 13 families, highlighting the potential of bryophytes as a source of bioactive compounds associated with the cholinergic system [21], thus establishing a relevant precedent for the investigation of *S. magellanicum*. Beyond their pharmacological potential, the ethnomedicinal use of *Sphagnum* species across diverse cultures, particularly for wound care, infection treatment, and fever management, provides a traditional basis for the scientific investigation of their bioactivity [1,2].

In the present study, we report for the first time the chemical composition and AChE inhibitory activity of the volatile fraction obtained by steam distillation from the aerial parts of *S. magellanicum* Brid. GC-MS and GC-FID were used to analyze the volatile fraction, and the anticholinesterase potential was evaluated using an in vitro Ellman assay.

## 2. Results

### 2.1. Volatile Fraction Yield and Chemical Composition

**Figure 1:**
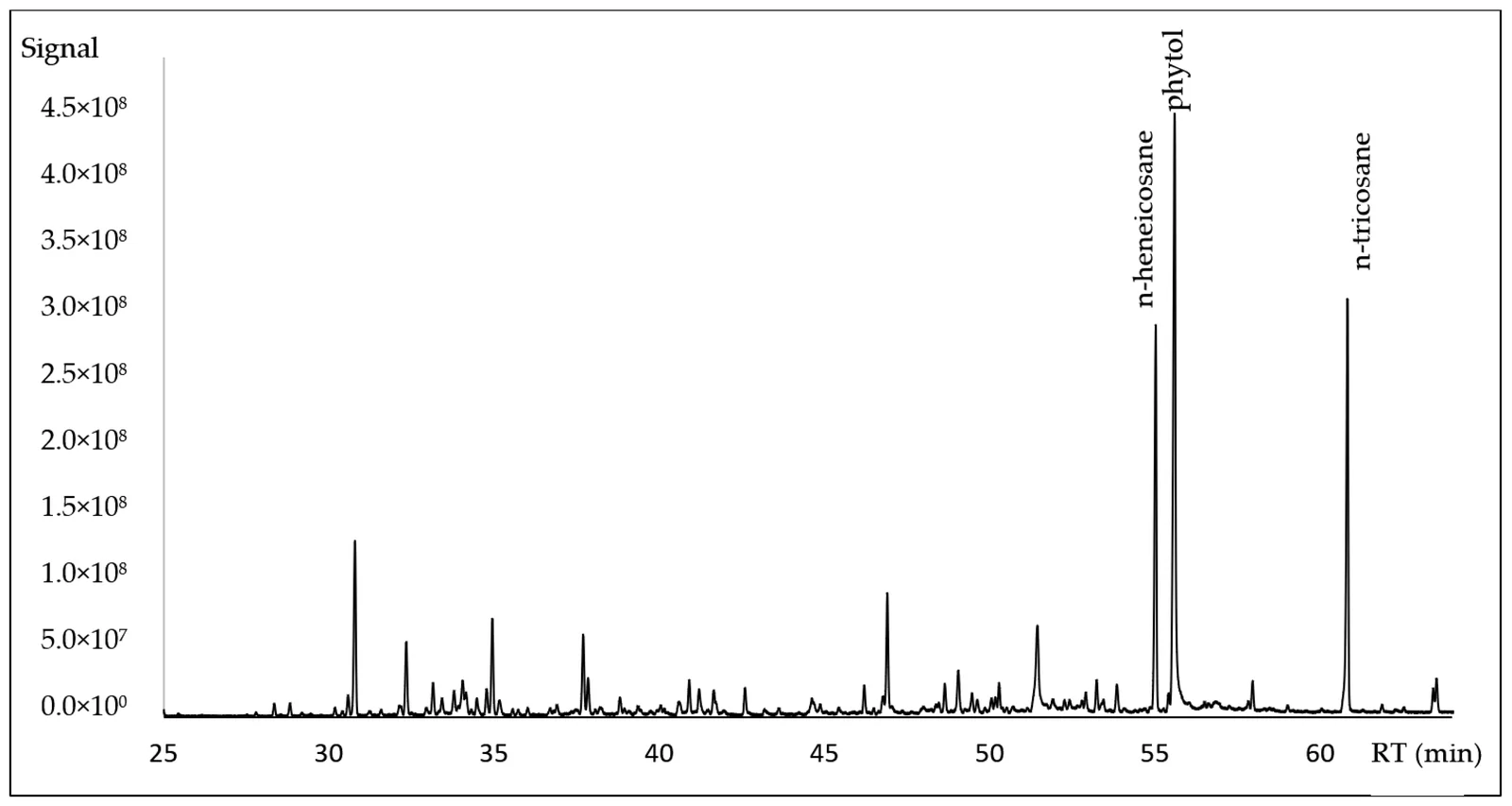
GC-MS total ion chromatogram (TIC) of the volatile fraction of *S. magellanicum* from Ecuador (TR-5MS column). Labelled peaks: phytol, n-heneicosane and n-tricosane.

The volatile fraction of the aerial parts of *S. magellanicum* Brid. was obtained by steam distillation, yielding an average of 0.032% (*w*/*w*). The GC-MS analysis using a DB-5ms capillary column (Figure 1) identified 89 compounds, which accounted for 93.03%, 96.41%, and 96.41% of the total volatile fraction in each replicate, respectively. The complete qualitative and quantitative composition was presented in Table 1. We classified the identified compounds into twelve chemical classes. Alkanes were the dominant group, accounting for 39.94% ± 0.39%. The second most abundant class was alcohols (17.56% ± 0.30%), followed by sesquiterpene hydrocarbons (13.10% ± 1.15%), oxygenated sesquiterpenes (6.10% ± 0.89%), aldehydes (4.56% ± 1.22%), and fatty acids (4.05% ± 0.02%). Minor classes included ketones (3.39% ± 0.25%), diterpenes (2.15% ± 0.27%), esters (1.17% ± 0.20%), lactones (0.72% ± 0.31%), and monoterpene hydrocarbons (0.20% ± 0.02%).

The eleven most abundant compounds (>2.0% mean content), which accounted for approximately 64.88% of the total identified fraction, were summarized in Table 2. Phytol was identified as the dominant compound (16.09% ± 0.07%), followed by n-tricosane (9.25% ± 0.82%), n-heneicosane (9.17% ± 0.54%), hexacosane (7.30% ± 0.99%), nonacosane (5.42% ± 0.14%), (Z)-caryophyllene (4.08% ± 0.32%), hexadecanoic acid (3.64% ± 0.25%), and dotriacontane (2.93% ± 0.21%).

### 2.2. Acetylcholinesterase Inhibitory Activity

**Figure 2:**
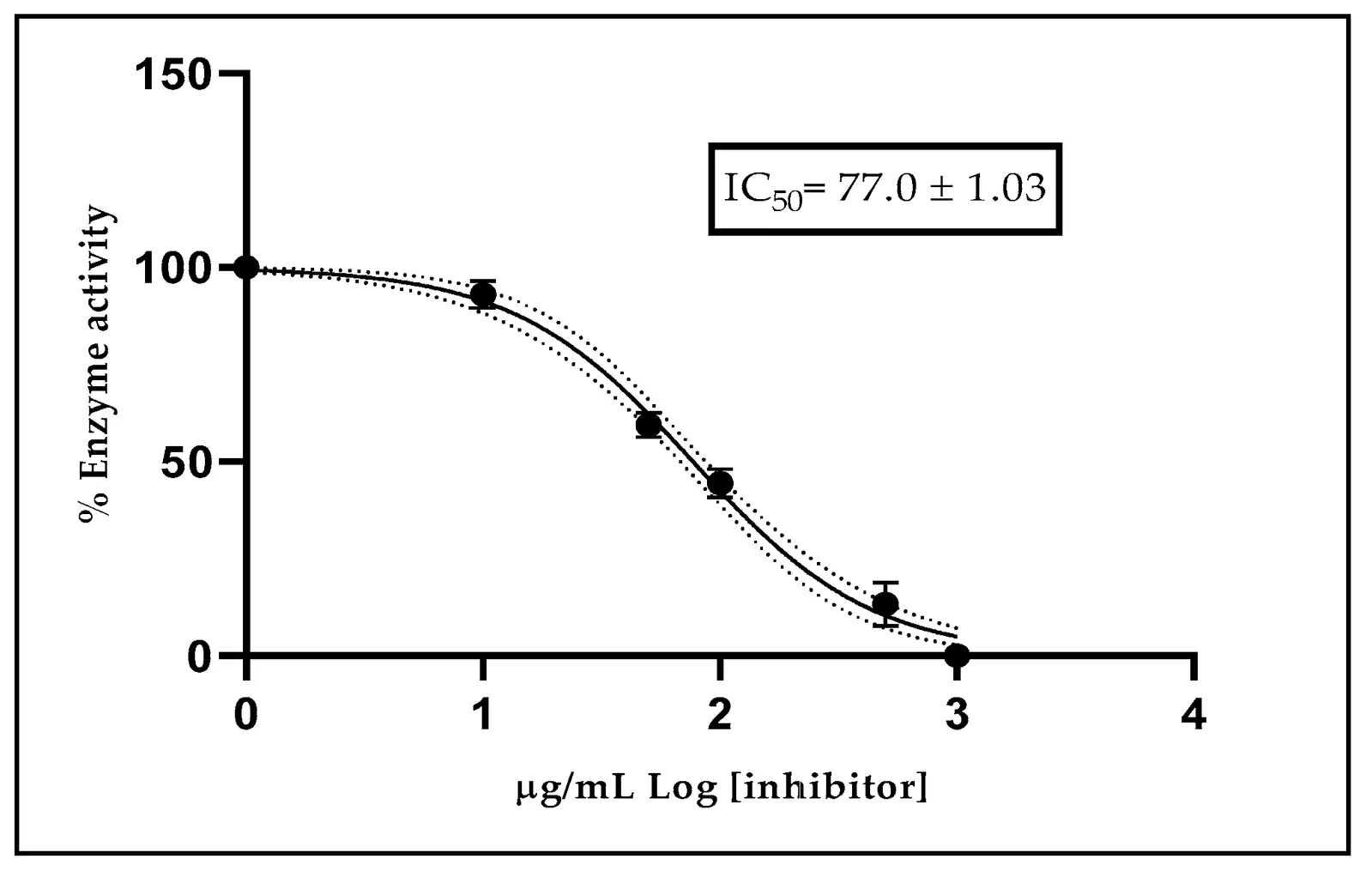
AChE inhibitory activity of the volatile fraction of *S. magellanicum* from Ecuador. The IC_50_ values were calculated from concentration-response curves (mean ± SD, n = 3).

The VF of *S. magellanicum* was evaluated for its AChE inhibitory potential using the Ellman colorimetric assay. Figure 2 shows the concentration-response curve. The volatile fraction exhibited moderate AChE inhibitory activity, with an IC_50_ value of 77.0 ± 1.03 µg/mL. For comparison, the IC_50_ of donepezil hydrochloride (12.40 ± 1.35 nM; molar mass 415.96 g/mol) corresponds to approximately 5.2 ± 0.6 ng/mL (0.0052 µg/mL), indicating that the VF is about four orders of magnitude less potent on a mass-concentration basis. This comparison involves a complex mixture and a pure compound and is therefore not equivalent on a molar basis.

## 3. Discussion

The volatile fraction of *Sphagnum magellanicum* Brid. collected in southern Ecuador was characterized for the first time, revealing a profile of 89 identified compounds representing 93.03–96.41% of the total VF. The dominant chemical class was *n*-alkanes (39.94%), specifically *n*-heneicosane (C_21_, 9.17 ± 0.54%), *n*-tricosane (C_23_, 9.25 ± 0.82%), hexacosane (C_26_, 7.30 ± 0.99%), nonacosane (C_29_, 5.42 ± 0.14%), and dotriacontane (C_32_, 2.93 ± 0.21%). This predominance is consistent with the well-documented composition of the genus *Sphagnum*, in which *n*-alkane distributions in cuticle waxes are typically dominated by mid- to long-chain homologues (C_21_–C_33_), with C_23_ or C_25_ as the major homologues across multiple species and geographic regions [23,24]. In bryophytes, long-chain n-alkanes are structural components of cuticular waxes, playing a key role in waterproofing and desiccation tolerance [25]. These functions are particularly relevant for plants growing in areas with fluctuating humidity levels, such as the Andes and the Amazon [26]. The higher proportions of alkanes observed in our Ecuadorian material relative to European *Sphagnum* extracts [3] may thus reflect an adaptive response to the environmental stress conditions of the collection site at c.a. 2740 m a.s.l.

Phytol was the most abundant compound and a finding of considerable pharmacological significance [27]. As an acyclic diterpene alcohol derived from the phytol side chain of chlorophyll through hydrolysis and reduction [28], phytol is ubiquitous in photosynthetic biomass, and its occurrence in *Sphagnum* extracts has been documented previously, although not as a major constituent [3]. The broad pharmacological profile of phytol is well established: Islam et al. [27] demonstrated significant anti-inflammatory activity through inhibition of COX-1, COX-2, NF-κB, and IL-1β pathways in preclinical models, while Claro-Cala et al. [29] confirmed its anti-inflammatory effect on primary human monocytes-macrophages. A systematic meta-analysis confirmed a significant association between phytol and cytotoxicity in cancer cell models (OR: 1.81; 95% CI = 1.12–2.65, *p* < 0.05), supporting its anticancer potential [30]. Additionally, in silico studies suggest that phytol interacts with the AChE active site gorge through hydrophobic contacts with key residues (Trp86, Tyr337) [12,31]; however, its contribution to the AChE inhibition observed for the VF cannot be inferred from the present data.

The sesquiterpene fraction comprising (*Z*)-caryophyllene (4.08 ± 0.32%), δ-amorphene (2.15 ± 0.19%), α-humulene (2.01 ± 0.18%), caryophyllene oxide (1.79 ± 0.17%), and germacrene D (0.65 ± 0.06%) contributes an additional pharmacological dimension to the VF. Caryophyllene sesquiterpenes are widely recognized for their genoprotective, cytoprotective, antiproliferative, and chemosensitizing activities [32]. The presence of a sesquiterpene fraction exceeding 19% in Ecuadorian specimens of *Sphagnum magellanicum*, in stark contrast to its absence in material from high-latitude regions of Europe [3], suggests a strong geographic and environmental influence on its lipid profile. The chemical profile of *S. magellanicum* of Baltic origin is characterized by low complexity and is dominated by fatty acids, sterols and triterpenes. No sesquiterpenes were detected in its lipid fraction [3]. This marked variation in chemical composition can be explained by the stress induced by Andean-Amazonian conditions (temperature fluctuations and high UV radiation), which stimulate the accumulation of terpenoids as cellular photoprotectors [33]. In bryophytes, the regulation and dynamics of sesquiterpene synthesis in response to abiotic stress primarily depend on the activity of microbial-type terpene synthases (MTPSLs) [33].

Hexadecanoic acid (palmitic acid, 3.64 ± 0.25%), the dominant fatty acid in the VF, was previously reported as a biologically active constituent of multiple *Sphagnum* species analyzed by GC-MS [6]. Structurally, hexadecanoic acid acts as a competitive inhibitor of phospholipase A_2_ (PLA_2_), the enzyme initiating prostaglandin and leukotriene biosynthesis, thereby conferring anti-inflammatory properties validated by crystallographic and in vivo data [34,35]. Its co-occurrence with phytol and (*Z*)-caryophyllene in the VF raises the possibility of additive anti-inflammatory effects, a synergistic phenomenon documented for complex plant volatile fractions [15,16].

The volatile profile of *S. magellanicum* from Ecuador differs markedly from those reported for other bryophytes by steam distillation. Sim-Sim et al. [11] reported predominantly oxygenated sesquiterpene profiles for *S*. *palustre* and *S. magellanicum* from Portugal, with no dominant diterpene alcohol. These interspecific and interregional contrasts reflect the well-documented influence of geographic origin, altitude, season, and extraction methodology on the secondary metabolite profiles of bryophytes [5] and underscore the chemical distinctiveness of the Ecuadorian chemotype described here. The volatile fraction was obtained by steam distillation, which provides material suitable for bioassays, but which may not fully reflect the composition of the headspace of living moss and may generate thermal artefacts. Complementary techniques, such as headspace solid-phase microextraction (HS-SPME), would enable the comparison of profiles obtained using different extraction methods, and are proposed for future work.

The VF of *S. magellanicum* demonstrated moderate AChE inhibitory activity (IC_50_ = 77.0 ± 1.03 µg/mL), the first report of this biological activity for the species. While this value is considerably higher than that of the synthetic reference donepezil (IC_50_ = 0.0052 µg/mL). In the regional context, the VF of the Ecuadorian liverwort *Syzygiella rubricaulis* showed a stronger inhibitory effect (IC_50_ = 26.75 ± 1.03 µg/mL), which may be related to its markedly distinct sesquiterpene-dominated profile (hydrocarbon sesquiterpenes: 48.35%; oxygenated sesquiterpenes: 46.89%) [36]. This comparison is in line with reports suggesting a relationship between sesquiterpene content and AChE inhibitory potency: Venditti et al. [16] suggest that an increase in sesquiterpenoid proportion relative to monoterpenoids considerably improves AChE inhibition capacity, and Hung et al. [15] confirmed that VFs with high sesquiterpene concentrations systematically show stronger activity. The moderate AChE inhibitory activity observed for *S. magellanicum* may be related to its chemical profile, which is dominated by *n*-alkanes and alcohols, compounds for which AChE inhibitory activity appears to be highly dependent on their specific chemical structures and concentrations [37,38].

The whole-fraction assay used here does not allow the contribution of individual constituents to the observed AChE inhibition to be determined, and no attribution of the activity to specific compounds. Previous studies on EOs and VFs have reported cholinesterase-inhibitory effects for some terpenoid constituents, including sesquiterpenes such as caryophyllene. However, these effects are highly dependent on molecular structure, concentration, and assay conditions [12,15,17,37,39]. The present data do not allow us to determine whether the terpenoid constituents present in this VF (e.g., phytol and caryophyllane-type sesquiterpenes) participate in the activity measured here. Similarly, synergistic or additive interactions, which have been suggested for complex volatile fractions [15,16], cannot be assessed with a single whole-fraction assay and should be evaluated using a single whole-fraction assay and are therefore hypothetical. Bioassay-guided fractionation and testing of isolated constituents, individually and in combination, would be necessary to clarify their contribution.

## 4. Materials and Methods

### 4.1. Plant Material Collection and Preparation

Aerial parts of *Sphagnum magellanicum* Brid. (Sphagnaceae) were collected on May, June and July 2024 at Sector El Tiro, Sabanilla, Zamora Chinchipe province, southern Ecuador (latitude 3°58′05.9″ S, longitude 79°08′00.5″ W, altitude 2740 m a.s.l.). The collection was conducted under permit No. MAATE-ARSFC-2022-2839 issued by the Ministerio del Ambiente, Agua y Transición Ecológica (MAATE) of Ecuador. Botanical identification of the specimen was carried out by Dr. Ángel Benítez, botanist at the Herbarium of the Universidad Técnica Particular de Loja (HUTPL), Loja, Ecuador. A reference voucher specimen has been deposited at the HUTPL Herbarium (HUPTPL AB-1331).

To ensure uniformity in environmental growth conditions and population source, plant material was collected from the same specific plant community. Upon arrival at the laboratory, extraneous material and debris were manually removed. The plant material was ground using a mechanical mill to reduce particle size and maximize surface area, facilitating more efficient volatile release during steam distillation. The processed material was used immediately for volatile fraction extraction.

### 4.2. Isolation of the Volatile Fraction by Steam Distillation

A total of 1502.41, 1201.03, and 1302.50 g of processed plant material were distributed uniformly across three round-bottom flasks. The VF was obtained by steam distillation using a laboratory-adapted Dean–Stark apparatus, as previously described by Calva et al. [40] and Maldonado et al. [41]. The condensed VF was separated from the aqueous phase, dried over anhydrous sodium sulfate (Na^2^SO^4^), filtered, transferred to amber glass vials, and stored at −7 °C until analysis. The yield of the volatile fraction was calculated as the mean percentage (*w*/*w*) relative to the fresh weight of plant material used.

### 4.3. GC-MS Analysis

Qualitative identification of volatile fraction constituents was performed using the methodology of Calva et al. [40], gas chromatography coupled to mass spectrometry (GC-MS) using a Thermo Scientific TRACE 1310–ISQ™ 7000 chromatograph (Waltham, MA, USA) equipped with a TR-5MS capillary column (30 m × 0.25 mm i.d. × 0.25 µm film thickness, equivalent to DB-5ms). Samples were prepared by dissolving 10 µL of the volatile fraction in 990 µL of dichloromethane (DCM, analytical grade); each preparation was injected in duplicate. Injection volume was 1 µL in split mode at a split ratio of 1:20, with an injector temperature of 230 °C.

The oven temperature program was as follows: initial hold at 50 °C for 10 min; temperature ramp at 3 °C min^−1^ to 170 °C; ramp at 10 °C min^−1^ to 230 °C; ramp at 15 °C min^−1^ to 280 °C, held for 10 min; total run time 70 min. Helium was used as the carrier gas at a constant flow. The mass spectrometer was operated in electron ionization (EI) mode at 70 eV, with a mass transfer line temperature of 230 °C and an ion source temperature of 230 °C. The mass scan range was *m*/*z* 40–400, with a solvent delay of 3 min. Compound identification was carried out by comparing calculated linear retention indices (LRI_cal_) with literature reference values (LRI_lit_) from the Adams database [22], and by spectral matching against the NIST 2017 mass spectral library, with a minimum accepted match factor of 80%.

### 4.4. GC-FID Analysis

Quantitative composition of the volatile fraction was determined by gas chromatography with flame ionization detection (GC-FID) on the same chromatographic platform described in Section 4.3. Analyses were performed in split mode at a ratio of 40:1. The oven temperature program consisted of an initial hold at 50 °C for 3 min, followed by a linear ramp to 230 °C over 3 min. The relative percentage of each identified compound was calculated by peak area normalization against the total chromatogram area, without the application of response correction factors. Results are reported as the mean percentage ± standard deviation (% ± SD) from three independent sample preparations (M1, M2, and M3). Percentages represent relative peak areas (area normalization without response factors) and should be interpreted as relative composition rather than absolute concentrations. FID response differs between hydrocarbons and oxygenated compounds; oxygenated constituents such as phytol may therefore be modestly underestimated relative to hydrocarbons.

### 4.5. Calculation of Linear Retention Indices

Linear retention indices (LRI) were calculated for all detected peaks according to the Van Den Dool and Kratz method [42], using a homologous series of *n*-alkane standards (C9–C25) injected under identical chromatographic conditions as the volatile fraction samples. Compound identity was confirmed when both the LRI_cal_ and the mass spectral fragmentation pattern corresponded to the respective entry in the Adams reference database [21] and/or the NIST library. Compounds for which no adequate spectral or retention index match was obtained are reported as “not identified” in Table 1, together with their calculated LRI values.

### 4.6. Acetylcholinesterase Inhibitory Activity Assay

The AChE inhibitory activity of the volatile fraction was evaluated in vitro following the colorimetric Ellman method [43], with minor modifications as detailed below.

#### 4.6.1. Sample Preparation

A stock solution of the volatile fraction (10,000 µg/mL) was prepared by dissolving 10 mg of the VF in 1 mL of methanol (MeOH, HPLC grade). Serial dilutions were prepared in MeOH to yield working concentrations of 1000, 500, 100, 50, and 10 µg/mL. Methanol was selected as a non-selective protic solvent and employed as the negative control at a maximum final concentration of 10% (*v*/*v*) in the reaction mixture, a level previously confirmed to exert no significant effect on AChE enzymatic activity.

#### 4.6.2. Enzymatic Assay Procedure

The reaction mixture was assembled in a 96-well flat-bottom microplate as follows: 40 µL of Tris buffer (pH 8.0), 20 µL of the test sample at the corresponding working concentration, 100 µL of 5,5′-dithiobis-(2-nitrobenzoic acid) (DTNB, 3 mM in Tris buffer), and 20 µL of acetylthiocholine iodide (ATCh, 15 mM in phosphate-buffered saline, PBS, pH 7.4). The mixture was pre-incubated for 3 min at 25 °C with continuous agitation. The enzymatic reaction was initiated by adding 20 µL of acetylcholinesterase (AChE, 0.5 U/mL, from *Electrophorus electricus*, Sigma-Aldrich, St. Louis, MO, USA). The formation of the chromogenic product 5-thio-2-nitrobenzoate (TNB), resulting from the reaction of thiocholine with DTNB, was monitored spectrophotometrically at 405 nm using an EPOCH 2 microplate reader (BioTek Instruments, Winooski, VT, USA) at 25 °C over 60 min. A standard calibration curve of DTNB and L-glutathione (L-GSH) at multiple molar concentrations was constructed to convert absorbance readings to product release rates (mM min^−1^).

#### 4.6.3. Controls, IC_50_ Determination, and Data Analysis

Donepezil hydrochloride (Sigma-Aldrich, ≥98% purity) was employed as the positive reference AChEI. Under the same experimental conditions described above, donepezil yielded a calculated IC_50_ of 12.40 ± 1.35 nM, confirming assay system validity and reproducibility. The percentage of AChE inhibition was calculated according to Equation (1) [44]:

%Inhibition = (1 − Vsample/Vcontrol) × 100(1)

where *V*_sample_ and *V*_control_ represent the reaction velocities (mM min^−1^) measured in the presence and absence of the test sample, respectively. IC_50_ values were determined from concentration–response curves by nonlinear regression analysis (log[inhibitor] vs. normalized response, variable slope model) using GraphPad Prism software (version 9.0; GraphPad Software, San Diego, CA, USA) [45]. All assays were performed in triplicate.

### 4.7. Statistical Analysis

All quantitative data are expressed as mean ± standard deviation (SD) from three independent experimental replicates (n = 3). The relative percentage composition of the volatile fraction was determined by peak area normalization of GC-FID chromatograms. IC_50_ values for AChE inhibition were calculated by nonlinear regression analysis using GraphPad Prism v9.0 (GraphPad Software, San Diego, CA, USA). Statistical significance was set at *p* < 0.05.

## 5. Conclusions

The present study reports, for the first time, the chemical characterization and acetylcholinesterase inhibitory activity of the volatile fraction obtained by steam distillation from the aerial parts of *Sphagnum magellanicum* Brid. collected in southern Ecuador. A total of 89 compounds were identified, accounting for 93.03–96.41% of the total volatile fraction. The chemical profile of the volatile fraction was dominated by *n*-alkanes (39.94%), a finding consistent with the lipid geochemistry previously described for the genus *Sphagnum*, though the specific alkane distribution and the remarkable abundance of phytol (16.09 ± 0.07%) as the single most prevalent compound represent a distinctive feature of this Ecuadorian chemotype. Phytol and caryophyllene-type sesquiterpenes, which co-occur in the VF, have been associated in the literature with anti-inflammatory and antinociceptive activities [27,28,29,30,31,32]; their relevance to the AChE inhibition observed here was not evaluated. The volatile fraction demonstrated moderate acetylcholinesterase (AChE) inhibitory activity with an IC_50_ value of 77.0 ± 1.03 µg/mL in the Ellman in vitro assay, representing the first report of anticholinesterase potential for this species. This moderate activity may be related to the predominance of *n*-alkanes over sesquiterpenes in the VF, given that sesquiterpene-rich profiles are typically associated with higher AChE inhibitory potency. The results further validate the pharmacological relevance of Ecuadorian bryophytes as a largely untapped source of bioactive secondary metabolites and encourage expanded phytochemical investigation of the genus *Sphagnum* in the megadiverse Andean region.

## Figures and Tables

**Table 1:** Chemical composition of the volatile fraction of *Sphagnum magellanicum* Brid.

N°	Compound	LRI_cal_	LRI_Lit_	%			
				M1	M2	M3	% ± SD
1	myrcene	990	988	0.18	0.18	0.18	0.18 ± 0.00
2	1,8-cineole	1034	1026	0.02	-	-	0.01 ± 0.01
3	(Z)-β-ocimene	1039	1032	0.03	-	-	0.01 ± 0.02
4	γ-terpinene	1061	1054	-	0.02	0.02	0.01 ± 0.01
5	geraniol	1247	1252	0.05	0.04	0.04	0.05 ± 0.00
6	methyl nerolate	1275	1280	0.08	0.08	0.08	0.08 ± 0.00
7	bornyl acetate	1283	1284	0.13	0.15	0.15	0.15 ± 0.01
8	2-undecanone	1301	1293	0.07	0.07	0.07	0.07 ± 0.00
9	α-cubebene	1343	1348	0.02	0.02	0.02	0.02 ± 0.00
10	α-terpinyl acetate	1350	1346	0.09	0.09	0.09	0.09 ± 0.00
11	silphiperfol-5,7(14)-diene	1359	1358	0.26	0.31	0.31	0.29 ± 0.03
12	cyclosativene	1363	1369	-	0.03	0.03	0.02 ± 0.02
13	α-ylangene	1369	1373	0.3	0.36	0.36	0.34 ± 0.03
14	β-bourbonene	1377	1381	0.04	0.06	0.06	0.05 ± 0.01
15	(E)-β-damascenone	1381	1383	0.1	0.12	0.12	0.11 ± 0.01
16	2-dodecanone	1408	1397	0.24	0.28	0.28	0.27 ± 0.02
17	dodecanal	1413	1408	0.41	0.47	0.47	0.45 ± 0.04
18	(Z)-caryophyllene	1417	1408	3.71	4.26	4.26	4.08 ± 0.32
19	β-gurjunene	1428	1433	0.07	0.07	0.07	0.07 ± 0.00
20	aromadendrene	1437	1441	0.13	0.14	0.14	0.14 ± 0.01
21	alloaromadendrene	1441	1455	-	0.01	0.01	0.01 ± 0.01
22	α-himachalene	1446	1449	0.1	0.12	0.12	0.11 ± 0.01
23	α-humulene	1452	1454	1.81	2.11	2.11	2.01 ± 0.18
24	4,5-di-epi-aristolochene	1466	1473	0.11	0.13	0.13	0.13 ± 0.01
25	germacrene D	1472	1481	0.58	0.68	0.68	0.65 ± 0.06
26	γ-muurolene	1477	1479	0.35	0.41	0.41	0.39 ± 0.04
27	β-selinene	1485	1490	0.46	0.56	0.56	0.53 ± 0.06
28	β-macrocarpene	1491	1499	0.54	0.64	0.64	0.61 ± 0.06
29	2-tridecanone	1503	1496	0.01	0.03	0.03	0.02 ± 0.01
30	β-bisabolene	1509	1505	0.42	0.5	0.5	0.47 ± 0.05
31	γ-cadinene	1515	1513	0.51	0.59	0.59	0.56 ± 0.05
32	δ-amorphene	1519	1512	1.93	2.26	2.26	2.15 ± 0.19
33	cis-calamenene	1525	1529	0.13	0.15	0.15	0.14 ± 0.01
34	trans-cadina-1,4-diene	1534	1534	0.12	0.14	0.14	0.13 ± 0.01
35	α-cadinene	1537	1538	0.11	0.13	0.13	0.12 ± 0.01
36	α-calacorene	1544	1545	0.11	0.13	0.13	0.12 ± 0.01
37	silphiperfol-5-en-3-ol A	1554	1559	0.04	0.04	0.04	0.04 ± 0.00
38	germacrene B	1558	1561	0.06	0.08	0.08	0.07 ± 0.01
39	(E)-nerolidol	1566	1563	0.3	0.41	0.41	0.37 ± 0.06
40	n-tridecanol	1569	1571	0.17	0.24	0.24	0.22 ± 0.04
41	caryophyllene oxide	1583	1583	1.6	1.88	1.88	1.79 ± 0.17
42	gleenol	1587	1587	0.74	0.87	0.87	0.83 ± 0.08
43	guaiol	1601	1600	0.15	0.18	0.18	0.17 ± 0.01
44	humulene epoxide ii	1616	1608	0.16	0.18	0.18	0.17 ± 0.01
45	tetradecanal	1620	1612	0.22	0.27	0.27	0.25 ± 0.03
46	epi-α-cadinol	1631	1640	0.26	0.29	0.29	0.28 ± 0.02
47	α-muurolol	1641	1646	0.09	0.1	0.1	0.10 ± 0.01
48	caryophylla-4(12),8(13)-dien-5β-ol	1644	1640	-	0.08	0.08	0.06 ± 0.05
49	pogostol	1648	1653	0.06	0.08	0.08	0.08 ± 0.01
50	α-cadinol	1662	1654	0.48	0.57	0.57	0.54 ± 0.06
51	1-tetradecanol	1677	1676	0.78	0.94	0.94	0.89 ± 0.09
52	β-bisabolol	1681	1675	0.37	0.46	0.46	0.43 ± 0.06
53	epi-α-bisabolol	1690	1684	0.84	1.00	1.00	0.95 ± 0.09
54	2-pentadecanone	1705	1697	0.06	0.08	0.08	0.07 ± 0.01
55	pentadecanal	1722	1713	0.6	0.71	0.71	0.67 ± 0.07
56	β-davanone-2-ol	1734	1725	0.02	0.02	0.02	0.02 ± 0.00
57	mint sulfide	1744	1741	0.31	0.35	0.35	0.33 ± 0.02
58	8-hydroxy-dihydro-eremophilone	1753	1757	0.02	0.02	0.02	0.02 ± 0.00
59	n-pentadecanol	1766	1774	0.05	0.04	0.04	0.04 ± 0.01
60	(Z)-α-santalol acetate	1778	1778	-	0.65	0.65	0.43 ± 0.37
61	1-octadecene	1783	1790	1.34	0.3	0.3	0.64 ± 0.60
63	khusinol acetate	1813	1823	0.06	0.07	0.07	0.07 ± 0.01
64	2,7(14),10-bisabolatrien-1-ol-4-one	1823	1845	0.09	0.67	0.67	0.48 ± 0.33
65	2-pentadecanone, 6,10,14-trimethyl-	1837	1847	2.61	2.96	2.96	2.84 ± 0.21
66	n-hexadecanol	1862	1875	0.06	0.21	0.21	0.16 ± 0.09
67	epi-laurenene	1904	1901	1.07	1.24	1.24	1.18 ± 0.10
68	isophytol	1929	1947	1.3	1.52	1.52	1.45 ± 0.13
69	cyclohexadecanolide	1939	1934	-	0.04	0.04	0.02 ± 0.02
70	ent-rosa-5,15-diene	1940	1934	0.53	0.6	0.6	0.58 ± 0.04
71	hexadecanoic acid	1948	1960	3.36	3.78	3.78	3.64 ± 0.25
72	hexadecyl acetate	2015	2010	0.47	0.09	0.09	0.22 ± 0.21
73	7-hydroxy-4,8-dimethyl-coumarin	2034	2013	0.37	0.9	0.9	0.72 ± 0.31
74	kaurene	2053	2042	0.62	0.75	0.75	0.71 ± 0.08
75	n-heneicosane	2086	2100	8.55	9.49	9.49	9.17 ± 0.54
76	phytol	2120	2122	16.01	16.13	16.13	16.09 ± 0.07
77	9,12-octadecadienoic acid (Z,Z)-	2164	2170	0.68	0.28	0.28	0.41 ± 0.23
78	n-docosane	2201	2200	1.14	0.99	0.99	1.04 ± 0.09
79	(E)-phytol acetate	2220	2218	0.11	0.12	0.12	0.12 ± 0.00
80	n-tricosane	2299	2300	8.31	9.72	9.72	9.25 ± 0.82
81	henicosanal	2321	2329	3.48	0.6	0.6	1.56 ± 1.66
82	n-tetracosane	2408	2400	1.16	1.31	1.31	1.26 ± 0.09
83	n-pentacosane	2497	2500	2.99	1.17	1.17	1.78 ± 1.05
84	docosanal	2450	2434	1.25	1.81	1.81	1.62 ± 0.32
85	hexacosane	2600	2600	8.45	6.73	6.73	7.30 ± 0.99
86	heptacosane	2705	2700	0.45	0.59	0.59	0.54 ± 0.08
87	octacosane	2808	2800	-	0.87	0.87	0.58 ± 0.50
88	nonacosane	2901	2900	5.26	5.5	5.5	5.42 ± 0.14
89	dotriacontane	3201	3200	2.69	3.06	3.06	2.93 ± 0.21
	Total identified (%)			93.03	96.41	96.41	
	Alkanes (%)			40.34	39.72	39.72	
	Alcohols (%)			17.21	17.74	17.74	
	Sesquiterpene hydrocarbons (%)			12.94	15.14	15.14	
	Aldehydes (%)			5.96	3.86	3.86	
	Oxygenated sesquiterpenes (%)			5.36	7.04	7.04	
	Fatty acids (%)			4.03	4.06	4.06	
	Ketones (%)			3.1	3.54	3.54	
	Diterpenes (%)			1.92	2.27	2.27	
	Esters (%)			0.94	1.29	1.29	
	Lactones (%)			0.37	0.9	0.9	
	Monoterpene hydrocarbons (%)			0.21	0.20	0.20	
	Oxygenated Monoterpenes (%)			0.07	0.04	0.04	

LRI_cal_: calculated linear retention index; LRI_lit_: literature retention index according to Adams [22]; %: mean relative percentage; SD: standard deviation; n.d.: not detected.

**Table 2:** Major compounds (>2.0%) in the volatile fraction of *S. magellanicum* Brid.

Compound	Class	% ± SD
Phytol	Diterpene alcohol	16.09 ± 0.07
*n*-Tricosane	Alkane	9.25 ± 0.82
*n*-Heneicosane	Alkane	9.17 ± 0.54
Hexacosane	Alkane	7.30 ± 0.99
Nonacosane	Alkane	5.42 ± 0.14
(*Z*)-Caryophyllene	Sesquiterpene hydrocarbon	4.08 ± 0.32
Hexadecanoic acid	Fatty acid	3.64 ± 0.25
Dotriacontane	Alkane	2.93 ± 0.21
2-Pentadecanone, 6,10,14-trimethyl-	Ketone	2.84 ± 0.21
δ-Amorphene	Sesquiterpene hydrocarbon	2.15 ± 0.19
α-Humulene	Sesquiterpene hydrocarbon	2.01 ± 0.18

## Data Availability

The data generated by this research are presented throughout the manuscript. If you have any questions, please contact the corresponding author.

## Abbreviations

The following abbreviations are used in this manuscript:

VF	Volatile Fraction
GC-FID	Gas chromatography with Flame Ionization Detector
GC-MS	Gas chromatography coupled to mass spectrometry
AChE	Acetilcholinesterase
